# Colorectal Adenocarcinoma Cell Culture in a Microfluidically Controlled Environment with a Static Molecular Gradient of Polyphenol

**DOI:** 10.3390/molecules26113215

**Published:** 2021-05-27

**Authors:** Roman G. Szafran, Kazimierz Gąsiorowski, Benita Wiatrak

**Affiliations:** 1 Department of Biochemistry, Molecular Biology and Biotechnology, Faculty of Chemistry, Wroclaw University of Science and Technology, ul. Norwida 4/6, 50-373 Wroclaw, Poland; 2 Department of Basic Medical Sciences, Faculty of Pharmacy, Wroclaw Medical University, Borowska 211, 50-556 Wroclaw, Poland; kazimierz.gasiorowski@umed.wroc.pl (K.G.); benita.wiatrak@umed.wroc.pl (B.W.); 3 Department of Pharmacology, Faculty of Medicine, Wroclaw Medical University, Mikulicza-Radeckiego 2, 50-345 Wroclaw, Poland

**Keywords:** cancer, tumor-on-a-chip, microfluidic device, curcumin, *trans*-resveratrol, wogonin, LoVo cell line, static gradients, molecular gradients, tumor microenvironment, cell culture in gradients

## Abstract

To study the simultaneous effect of the molecular gradient of polyphenols (curcumin, *trans*-resveratrol, and wogonin) and biological factors released from tumor cells on apoptosis of adjacent cells, a novel microfluidic system was designed and manufactured. The small height/volume of microfluidic culture chambers and static conditions allowed for establishing the local microenvironment and maintaining undisturbed concentration profiles of naturally secreted from cells biochemical factors. In all trials, we observe that these conditions significantly affect cell viability by stimulating cell apoptosis at lower concentrations of polyphenols than in traditional multiwell cultures. The observed difference varied between 20.4–87.8% for curcumin, 11.0–37.5% for resveratrol, and 21.7–62.2% for wogonin. At low concentrations of polyphenols, the proapoptotic substances released from adjacent cells, like protein degradation products, significantly influence cell viability. The mean increase in cell mortality was 38.3% for microfluidic cultures. Our research has also confirmed that the gradient microsystem is useful in routine laboratory tests in the same way as a multiwell plate and may be treated as its replacement in the future. We elaborated the new repetitive procedures for cell culture and tests in static gradient conditions, which may become a gold standard of new drug investigations in the future.

## 1. Introduction

All living cells can sense their environment [1]. Concentration gradients of molecules play a crucial role in living organisms controlling multiple processes, including morphogenesis, wound healing, immune response, vessel pathfinding, and axonal guidance [2]. The concentration gradients of biomolecules are also responsible for cell differentiation and migration. A human body is built of about 10^13^ cells making up tissues and organs. Cells are surrounded by capillaries, which supply oxygen and nutrients to them. The capillaries also ensure the efficient delivery of most drugs. Cells in tissue are exposed to a concentration gradient of biologically active substances that penetrate from the blood into the cell’s environment. Depending on the distance of a particular cell from the delivery point, some cells are exposed to high and others to low concentrations of the substance, so its metabolic and/or phenotypic effects may differ. Cells exposed to a bioactive substance can secrete different amounts of another proapoptotic substance into their environment. It is, therefore, a complicated system in which the total effect of a compound on a group of cells in the tissue depends on: (1) the concentration of this compound that affects a particular cell, (2) local differences in the concentration of proapoptotic substances released from the cells that affect neighboring cells in the tissue. Pathologically degenerated tissues such as tumors form their complex microenvironment that differs significantly from the normal one in which gradients of oxygen, pH, nutrients, and metabolites are known to cause significant differences in cells’ proliferation, apoptosis rate, and their responses to chemotherapeutic agents [3,4]. Also, circulating DNA (cfDNA), a cell-free nucleic acid produced from dead, necrotic, and living cells, can affect neighboring cells. cfDNA fragments could enter neighboring cells and can alter the biology of the recipient. In tumors, they are involved in horizontal gene transfer and oncogenic transformation of normal cells and metastasis development [5].

Nowadays, the number of new drugs introduced to the pharmaceutical market does not correspond to the amount of funds allocated to develop new therapeutics [6]. This large imbalance is caused by the huge number of drug substances that fail in the late stages of preclinical or clinical trials, generating huge research costs. In the preliminary studies of new drugs, many in vitro research methods are used, from which the most common are monolayer cultures in multiwell plates. However, these tests tend to assess only the effect of the given substance concentration on cell molecular mechanisms, neglecting completely influences of molecular gradients present in tissues on the cell’s biology. In the search for novel therapeutics, to lower its costs and shorten the time, the use of more physiologically relevant experimental models that can mimic key aspects of the tissue microenvironment in vitro is required [7]. Recent advances in microfabrication techniques and tissue engineering have enabled the development of a wide range of 3D cell models. These include multicellular spheroids, organoids, scaffolds, hydrogels, organs-on-chips, and 3D bioprinting [8]. Although 3D cell cultures better mimic in vivo microenvironments of human tissues, they are also limited by having relatively low throughput and high unit cost. This is caused by complicated procedures of 3D model preparation that are time-consuming, laborious, and not standardized [9]. The laboratory use of 3D models requires trained lab staff and dedicated equipment and creates challenges for microscopy analysis and measurement. All this together cause that 3D models still cannot advance from research laboratories to industry.

Microfluidic systems have emerged as a potential way of recreating important aspects of the tumor microenvironment in vitro [10,11]. These systems have been used to investigate cellular processes such as tumor cell chemotaxis, angiogenesis, tumor cell extravasation, tumor-stroma cross-talk and cellular responses to drugs [12]. Conducting cell cultures in a lab-on-a-chip device allows taking into account the mutual interaction of cells simultaneously with the evaluation of the direct effect of the gradient of the active substance. The dimensions of the microsystem’s channels are usually in the same range as investigated biological systems (from several to several hundred micrometers), which ensures greater accuracy and control of the generated gradient in comparison to standard gradient culture systems, such as the Boyden chamber [13], the Zigmond chamber [14], the Dunn chamber [15], and micropipette-based assay [16,17]. Also, microfluidic systems operate on small volumes of liquid (nano- or microliters), so the research can be done with a smaller financial effort. A much smaller amount of biological and chemical waste is produced, which indirectly reduces environmental pollution with dangerous substances [18]. However, most microfluidic systems require highly specialized equipment and resources for microdevice fabrication (e.g., clean room, lithography or etching equipment) and operation (e.g., micropumps, microconnectors, microvessels). Microfluidic devices’ widespread adoption in biomedical and pharmacological research needs microdevices of simple construction adapted to the equipment and test procedures normally used in research and analytical laboratories [7].

Curcumin is one of the most frequently studied polyphenols of natural origin, whose beneficial effects on the human body are expected. It is a diarylheptanoid with a molecular weight of 369.38 g/mol isolated from the rhizomes of turmeric (*Curcuma longa* L.), a plant from the ginger family. It has been as possessing anti-inflammatory, antioxidant, antitumor, antimicrobial, supporting wound healing, anti-arthritic, anti-ischemic, anti-angiogenic, anti-mutagenic, anti-amyloid, and antidepressant activity [19,20]. In 2016, over 100 clinical trials were conducted, including the anticancer effects of curcumin and its use in cardiovascular diseases and inflammatory conditions such as arthritis and colitis. The second polyphenol that we have chosen for investigations was resveratrol. It is a stilbene with a molecular weight of 228.25 g/mol [21], isolated for the first time from the pollen hellebore in 1939 [22]. It is found in many plants and is produced by them as an anti-inflammatory substance in response to infection and oxidative stress. Resveratrol exists in the form of two isomers, *cis*- and *trans-*, but only the latter exhibits biological activity [23]. Resveratrol has a wide spectrum of pharmacological properties: antifungal, antibacterial, anti-inflammatory, cardio- and neuroprotective, anticancer, anti-aging, and protects against oxidative stress and antidiabetic [24,25]. The third substance chosen for tests was wogonin. It is a flavonoid with a 284.27 g/mol molecular weight, isolated from the root of *Scutellaria baicalensis* Georgi [26,27]. Flavonoids are the most abundant and physiologically active group of polyphenols. Many studies have confirmed that wogonin has a wide range of pharmacological properties including anti-inflammatory, anticancer, anti-anxiety, anti-atherosclerotic, anti-allergic, anti-thrombotic, anti-viral and antioxidant effects [28]. All the abovementioned polyphenols have undergone different stages of clinical trials. Looking at the results, they do not correlate with those expected from the previous preclinical tests. Potential discrepancies in the results between in vitro and in vivo tests and clinical trials may be caused by the low complexity of the cellular models used and also due to their low bioavailability in the human body.

Our study uses a gradient microdevice to analyze the impact of polyphenols concentration gradient on colorectal adenocarcinoma cells induced apoptosis. We chose three polyphenols, which antitumor activity was extensively studied in standard conditions-curcumin, *trans*-resveratrol, and wogonin. We assume that the induced gradient of polyphenol and the microenvironment self-arising in a chip culture microchamber influence cell chemosensitivity, same as observed for drug response [29,30]. We also want to show that our new microsystem is useful in routine laboratory tests in the same way as a multiwell plate, without the need for extra equipment, staff training, additional time consumption, and costs generation. In this article, we describe the new repetitive procedures of cell seeding, gradient generation, cell culture, and apoptosis detection, which may become in the future a gold standard for drug tests in molecular gradients.

## 2. Results and Discussion

### 2.1. Lab-on-a-Chip Device

**Figure 1:**
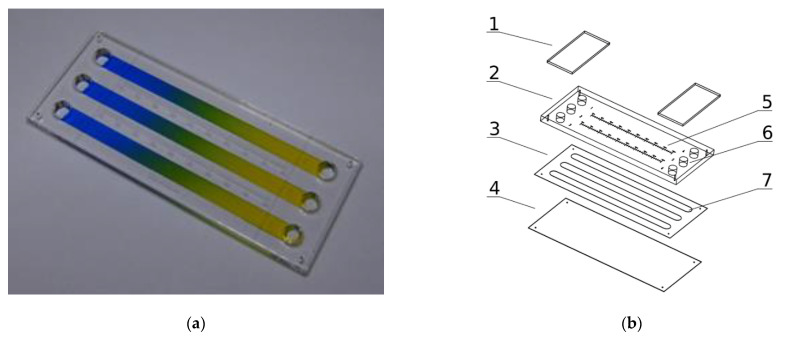
Microsystem for cell cultures in molecular gradients: (**a**) Photograph of a device with established gradients; (**b**) Exploded view of microsystem layers: 1—covers of media reservoirs, 2—polymethyl methacrylate layer, 3—adhesive film layer, 4—polycarbonate layer, 5—millimeter graduation, 6–media reservoirs, 7—culture chambers.

The microsystem that has been used for cell culture, with 75.9/31.2/3.33 mm (W/H/D) dimensions is presented in Figure 1a,b. It is composed of three layers: the bottom (4) made with transparent and elastic polycarbonate sheet of 0.2 mm thickness, the upper (2) made of a transparent polymethyl methacrylate sheet of 3 mm thickness, and the intermediate (3) made with a biocompatible adhesive film (7955MP, acrylic adhesive 200MP3M, St. Paul, MN, USA) of 0.13 mm thickness. The intermediate layer joins the upper and bottom layers and provides space for three culture chambers (7).

The dimensions of each chamber are 62.7/4.1/0.13 mm (W/H/D). The chambers are connected on both ends with the media reservoirs (6). Self-sticky covers cover the reservoirs (1) made with clear polyester sheets (Microseal ‘B’ PCR plate sealing film, Bio-Rad, Hercules, CA, USA). All the chip construction materials were cut or engraved using a commercial CO2 laser marker (Versa Laser VLS 2.30, Universal Laser System Inc., Scottsdale, AZ, USA) equipped with a 30 W CO2 (wavelength 10.6 µm) pulsed laser source equipped with a high power density focusing optic (HPDFO). The laser ablation of plastics is a fast and direct method perfectly suited for the fabrication of prototypes of high-quality microfluidic chips and short series production. It scales up to dozens of pieces a day, so it is well suited for lab-scale production. The microsystem construction is PDMS-free, easy to assemble and does not need a clean-room facility for fabrication.

Our gradient microdevice has three chambers, two of which can be used for the culture of cells. The first may be used for the control culture and the second for the culture in a concentration gradient of the tested substance. The third chamber is used to determine the substance’s concentration at a given chamber location–it is being filled out with an indicator, usually water, or a medium solution of dye. Measurement of indicator substance concentration is carried out by the spectrophotometric method in a multiwell plate reader. This configuration prevents the cells from coming into contact with the indicator substance. The active substance can be tested at ultralow concentrations, which cannot be detected directly by the spectrophotometric method. The microfluidic device works in a stationary (non-flow) regime without exposing the cells to stress related to shear stresses induced by the flowing fluids, which could influence cells viability [31]. The stationary regime also helps to maintain undisturbed concentration profiles of naturally secreted biochemical factors from the cells.

The operation of the microsystem is adapted to procedures and devices normally used in research and analytical laboratories. A chip is as simple as a standard multiwell culture plate and needs only standard laboratory equipment (micropipette, incubator and multiwell plate reader). The static gradients are generated quickly and repetitively (in a few seconds) by convective fluid movements caused by alternating tilting of the microdevice. Our chip’s design, manufacturing, and operation make it a universal, robust and efficient tool for everyday laboratory research.

### 2.2. Seeding Cells in Chambers of the Microfluidic Device

**Figure 2:**
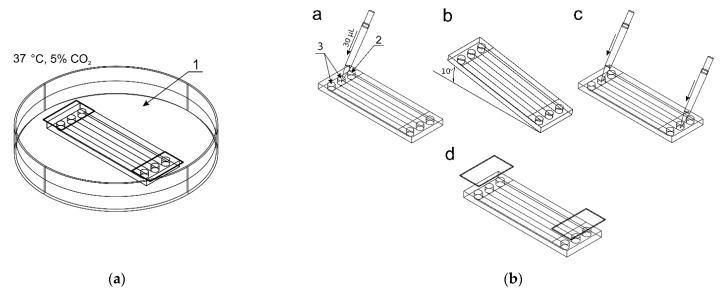
The procedure of seeding cells in chambers of the microfluidic device: (**a**) The microchip placed in a Petri dish with distilled water; (**b**) The seeding steps: a—adding a cell suspension to reservoirs of two culture chambers, b—directing the suspension to the chambers, c—final adding media, d—reservoirs protection.

Before starting the cultivation, the microsystem was sterilized by rinsing the chambers with 70% ethanol and then irradiated with UV light for 30 min. The culture chambers were filled with distilled water, the reservoirs secured with covers, and the microsystem placed in a Petri dish (1) partially filled with a distilled water (Figure 2a). The microdevice was then held for 72 h at 37 °C with a daily exchange of distilled water in the chambers to ensure continuous filling of the chambers and prevent air bubble formation. In the next stage, the cells were seeded into two culture chambers.

**Figure 3:**
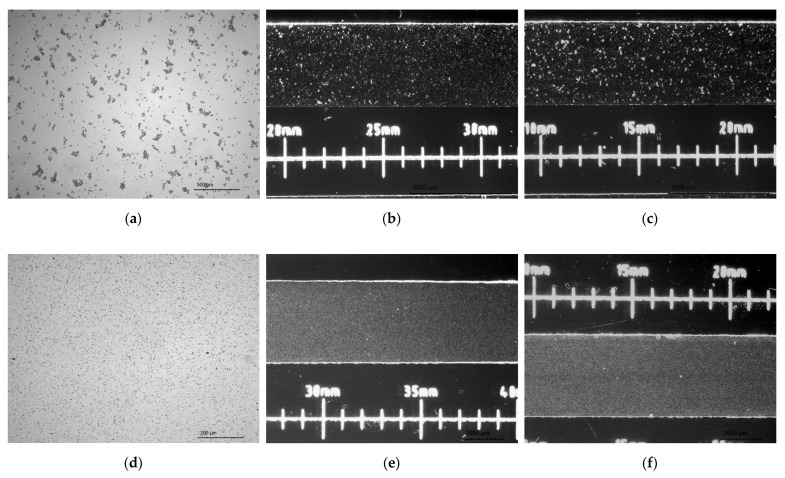
(**a**–**c**) Distributions of cells after seeding in a culture chamber; (**d**–**f**) Distributions of melamine microparticles after seeding in a culture chamber.

A 1 × 10^6^ cell suspension was prepared in 100 μL culture medium (per chamber). Figure 2b shows the process of seeding cells into the culture chambers of the chip. 30 μL of the cell suspension was added to inlets of two reservoirs (3) (control culture and the test culture) by a pipette. The indicator chamber (2) was filled with a complete medium. Then, the microchip was inclined to the constant angle of 10° to direct fluids from reservoirs to chambers. Thanks to this, during the slow flow of the cell suspension in the culture chamber, the cell sedimentation takes place, and they evenly cover the bottom surface of the chamber. The cell seeding process was repeated three times. The excess of fluids was removed from the outlet reservoirs each time. The distribution of cells in CC are presented in Figure 3a–c. Cells tend to agglomerate, which is a microdevice design-independent parameter and mainly depends on procedure of preparation of the suspension of cells prior to seeding. To reflect the device/procedure-dependent distribution parameter, we have also used melamine resins (cat. no. 90,637, Merck, Darmstadt, Germany) of 2 µm diameter as an indicator in seeding tests to investigate the uniformity of the CC surface coverage. The mean surface concentration of particles in the working area of culture chambers reached 6200 particles/mm^2^ with a standard deviation of 4.26% along a channel (Figure 3d,f). The value of SD was calculated from the analysis of microscopic photos taken along the chip’s CC with the interspace of 5 mm (10 × 3 = 30 pictures).

Finally, after seeding, a cell suspension or culture medium with a volume of approximately 30 μL (maximum capacity of the reservoirs) was appropriately added to reservoirs 2 and 3. The final stage of seeding cells was to secure the reservoirs with covers and place the microchips in a dish with distilled water in conditions of 5% CO_2_ and at 37 °C for 24 h for cells adhesion/immobilization.

### 2.3. Gradient Generation in Microdevice

**Figure 4:**
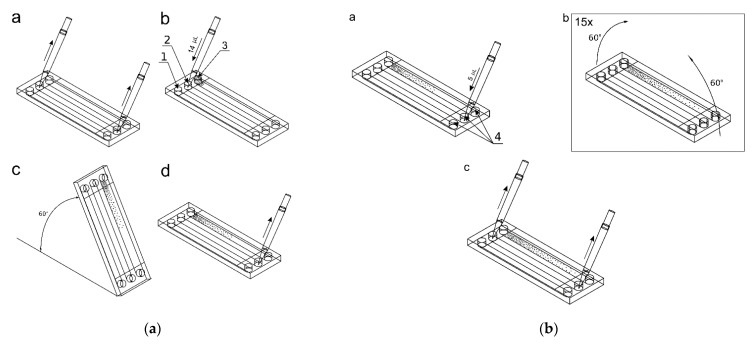
Setting the gradient of the active and indicator substances: (**a**) a—removing solutions from reservoirs, b—adding 14 μL of fluids, c—filling up the culture chambers, d—emptying excess fluids from the reservoirs; (**b**) a—addition of 5 μL of the complete medium, b—setting of gradients, c—emptying the reservoirs from the liquid.

After cells immobilization, removing of the reservoir’s covers and fluid removal from the inlet and outlet reservoirs, 14 μL of the active substance (50 μM/dm^3^ curcumin solution in culture medium, 100 μM/dm^3^ trans-resveratrol solution in culture medium or 75 μM/dm^3^ wogonin solution in culture medium) was added to the culture chamber 2 (Figure 4a). The same volumes (14 μL) of indicator dye (methylene blue at the concentration of 10 mM/dm^3^) and complete medium were added into the indicator chamber 1 and control culture chamber 3. These fluids were applied to specific chambers using appropriate inlet reservoirs and a micropipette. The microsystem was raised at an angle of 60° to introduce the solutions into the chambers, next the excess of solutions were removed from the outlet reservoirs, and 5 μL of the complete medium was added to the outlet reservoirs 4 (Figure 4a,b). In the next step, the gradients were prepared by tilting alternately of the microsystem by 60° once by once, repeating the process 15 times (Figure 4b). The repetitive tiling of the microsystem induces the reversed-direction transient flow responsible for convective transport of solute in a culture chamber, which causes the gradient generation of curcumin. Next, the excess of liquids was then removed from the reservoirs. The reservoirs were filled with 30 μL of corresponding substances within adjacent to the reservoir’s chambers (test compound solution, indicator solution and culture medium). After securing the reservoirs with covers, the measurement of absorbance value along an indicator chamber was made using a multiwell plate reader to evaluate the gradient of the indicator, which corresponds to the concentration gradient of the test compound. The microsystem was then placed in a dish with distilled water at 37 °C, 5% CO_2_. After a further 24 h, the gradient was measured in a spectrophotometer to check its stability.

After cells cultivation, the cell cultures in the culture chambers were stained with annexin V, and propidium iodide from the apoptosis kit, and a series of photos were taken in a fluorescence microscope at intervals of 5 mm.

### 2.4. Analysis of Cell Viability in Microdevice

**Figure 5:**
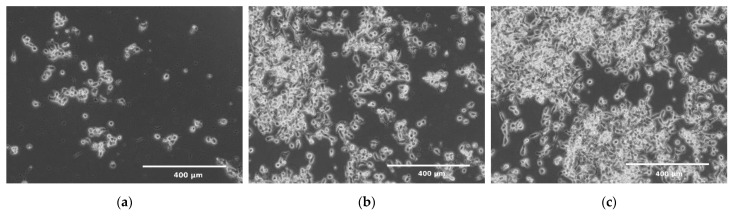

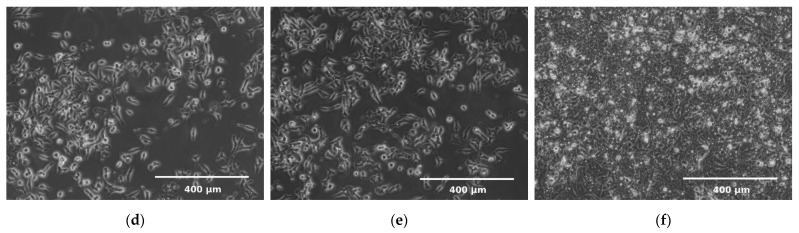
LoVo cells cultivation in conditions of a 5% CO_2_ and at 37 °C: (**a**) after 24 h in 96-well plate; (**b**) after 72 h in 96-well plate; (**c**) after 7 days in 96-well plate; (**d**) after 24 h in a microchip; (**e**) 72 h in a microchip; (**f**) after 7 days in a microchip.

It was necessary to examine whether the microsystem, particularly the materials from which it was made, had a negative effect on cell growth and differentiation. The viability of LoVo cells grown for 24 and 72 h at 5% CO_2_, 95% humidity at 37 °C in the microsystem culture chambers was evaluated. In parallel, traditional culture was carried out in a 96-well plate, which was a control. The viability was determined by the use of trypan blue, which stains only dead cells. After 24 and 72 h, the viability was determined to be 99.5 ± 0.5% (*n* = 4) and 99.3 ± 0.97% (*n* = 4) for cultures maintained in the microsystem and 99.9 ± 0.53% and 99.4 ± 1.45% for the control culture. This allows us to conclude that the microchip does not affect the viability of LoVo cells. Figure 5 compares the LoVo cell cultures in a multiwell plate and microfluidic device at different times in the culture.

### 2.5. Analysis of Cell Proliferation in Microdevice

**Figure 6:**
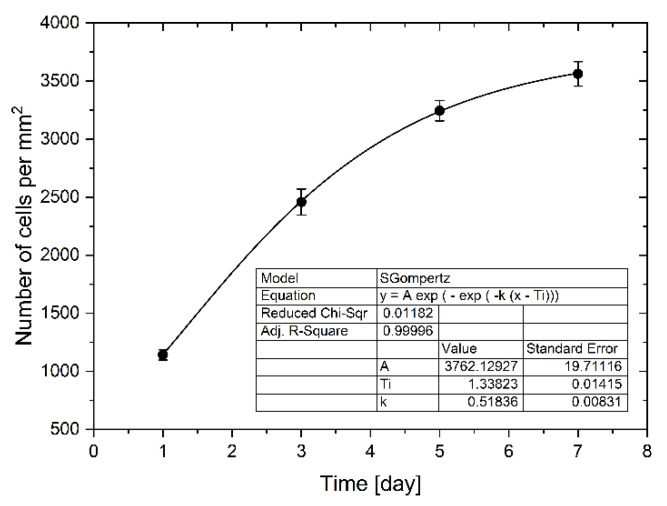
The LoVo cell proliferation in a microchip during weekly culture with every two days evaluation.

Cells proliferated and spread quickly on the culture chambers’ surface, increasing the degree of confluence. Between the first and the third day, an increase in proliferation was observed by about 115%. In the next days of cultivation 32% and 9% of the increase in proliferation was observed, respectively between 3 and 5 days and between 5 and 7 days of culture. Increased proliferation caused an increase in confluence, which resulted in slower cell division due to lack of space (Figure 6). The total convergence of the culture chambers’ surface increased during culture from 1% to 31%. We use the Gompertz model [32], a special case of the four parameter Richards model, to describe the LoVo cells proliferation in a microfluidic chip:

(1) $$W\left(t\right)=A\;\exp\left(-\exp\;\left(-k_{G}\left(t-T_{i}\right)\right)\right)$$

where *W(t)* is the number of cells as a function of time (days of cell cultivation), *t* is time, *A* represents the upper asymptote (adult value), *k_G_* is a growth-rate coefficient, and *T_i_* represents time at inflection. It is one of the most frequently used sigmoid models fitted to growth data and has been used, among other things, to describe tumor growth [33]. We found this model to be best describing cell proliferation in our gradient microfluidic chip. The determined parameters of the model are presented in Figure 6.

After subculturing, the cells were transferred from the microdevice to the traditional multiwell plate. The LoVo cells adhered to the surface of a well and continued to grow. This confirms that after cultivation in the microdevice, the cells retain the correct functional characteristics and the ability to grow in the subsequent traditional culture.

### 2.6. Gradient Repeatability and Stability Analysis

**Figure 7:**
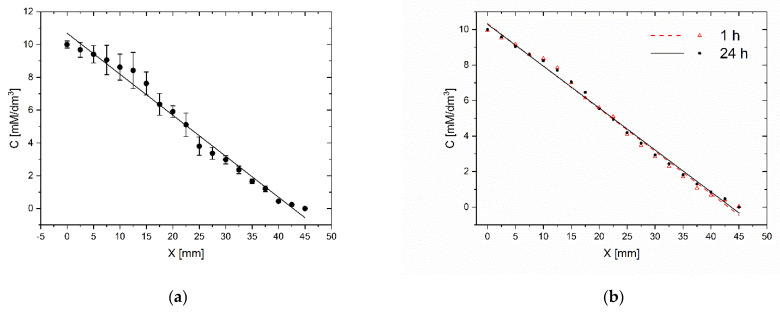
Evaluation of the gradient repeatability (**a**) and the gradient stability in time (**b**) C–methylene blue concentration, X–distance from the beginning of the workspace of CC. Evaluation immediately after establishment and after 24 h.

The repeatability of gradient generation and its stability in time are important factors that determine the practical use of the microsystem for daily laboratory tests. We carry out tests in five independent microsystems. The methylene blue dye’s gradient profile was determined by measuring the absorbance along the chamber containing the indicator. High repeatability of gradients was achieved for different chips. For the same gradient establishment procedure (15 tiles of the chip) and the same initial concentrations (10 mM), a similar dye concentration distribution along the indicator chamber was observed. The divergence of gradients between different chips was no more than 7% (Figure 7a). This parameter reflects the method’s susceptibility to human errors made while establishing the concentration gradient and the microsystem fabrication’s imprecision. The gradient generation procedure is not sensitive to differences in inclination angle of microsystem and time of tilting. The fluid flow in chambers is always stopped when fluid reservoirs are emptied. However, capillary forces prevent fluid from escape from chambers, even when the microsystem is situated at the right angle to the ground. Usually, it is not very important to have the same concentration profile in different chips. We always know the right concentration at each point of the culture chamber from dye absorbance measurement in the indicator chamber.

In the same study, the stability of the gradient was compared within 24 h. The absorbance was measured immediately after the gradient generation and after a 24-h incubation at 37 °C. The gradient changed by no more than 3% (Figure 7b). As the gradient generation procedure is based on the convective transport of molecules, the gradient profile and stability are not sensitive to the difference in the molecular weights of indicator and tested substance as long as the concentrations of the substances in the liquids filling the reservoirs do not change significantly.

### 2.7. Influence of Polyphenols Gradient on Cells’ Induced Apoptosis

To investigate the effect of the polyphenols concentration gradient and cellular interactions on the apoptosis of LoVo cells, studies were performed in a gradient microsystem and a traditional 96-well culture plate. The range of analysis of curcumin concentration in a microsystem was 50–10 μM, the concentration of resveratrol ranged from 100 to 15 μM and wogonin from 75 to 11 μM. Based on the assessment of apoptosis after 24-h incubation of LoVo cells with polyphenol, a curve of the dependence of the number of apoptotic cells on the concentration of each polyphenol was determined. Assessment of apoptosis was performed analogously in treating LoVo cell cultures with curcumin, *trans*-resveratrol, and wogonin concentrations in a multiwell plate.

Many studies have demonstrated the proapoptotic effects of tested compounds on colon cancer [34,35]. At the same time, it turned out that one of the mechanisms of anticancer activity that polyphenols, including curcumin, can influence is the excessive production of oxygen free radicals [34]. Curcumin is characterized by low bioavailability [36], therefore, in recent years, there has been a trend of looking for a method of delivering this compound in systems with various forms of drug carriers, e.g., liposomes, to increase systemic application possibility. In vitro studies showed that liposomal curcumin had a dose-dependent effect on LoVo cell viability in the MTT assay and increased cell number in apoptosis [36,37]. Like curcumin, trans-resveratrol also has low bioavailability. Therefore, the research was conducted in the context of exploiting its potential chemopreventive properties in colon cancer in vitro studies using the HT-29 cell line [35]. Like other polyphenols studied, Wogonin induces apoptosis in cancer cell lines by increasing ROS levels [38]. By inducing apoptosis, curcumin influences ultra-structural changes and thus the release of lactate dehydrogenase. Studies have also shown a reduction in mitochondrial membrane potential and activation of both caspase-3 and caspase-9.

Moreover, in the same cell line, an increase in the amount of released cytochromes c, Bax and p53 and a decrease in Bcl-2 in lysates of these cells were demonstrated, which confirms the proapoptotic effect on the mitochondrial-dependent pathway [39]. Moreover, curcumin stops the cell cycle in the S phase of LoVo cells. It has also been shown that curcumin activates AMP-activated 5 protein kinase (AMPK) and suppresses NF p65 phosphorylation-κB, uPA and MMP9 [36,40,41].

Similarly, *trans*-resveratrol induces apoptosis in colon cancer cells through an oxidative stress-dependent pathway, leading to DNA strand breakage [35]. After treatment of cell cultures with resveratrol, greater proapoptotic activity was observed in hematological tumors than in solid tumors. The proapoptotic effect of trans-resveratrol is associated with a reduction in Akt activation as a result of Ras downregulation, which facilitates the translocation of Bax into the mitochondria [42]. Resveratrol affects the mitochondria from which cytochrome c is released into the cytosol and disrupts the cellular redox state by producing ROS and lipid peroxidase. Subsequently, a deterioration in the GSH/GSSG ratio is observed.

Consequently, different classes of MAP kinases are preferentially activated in response to different oxidative stimuli (ROS versus GSH/GSSG) [43]. Also, wogonin causes oxidative stress on the endoplasmic reticulum. It induces the activation of caspase-9 and caspase-3 and up-regulates the expression of cleaved PARP [38]. There are changes in the ratio of anti-apoptotic/proapoptotic proteins of the Bcl-2 family and cleavage of Bid. Wogonin activates ERK and p38 MAPK by inhibiting N-acetylcysteine, increasing ROS and activating apoptosis [44,45].

As we see, the complex, synergistic mechanism of the proapoptotic and protective action of polyphenols, at the same time on normal and neoplastic cells, depends to a large extent on the properties of their growth environment where the biochemical factors are secreted at different amounts from cells. It is, therefore, a complicated system in which the total effect of a tested compound on a group of cells should be considered in conjunction with other factors. Failure to consider the influence of the environmental factors may lead to underestimating and overestimating the test results. Comparing the activity of the tested polyphenols in the microsystem and 96-well plate (Figure 5, Figure 6 and Figure 7), it can be seen that all examined compounds at the same concentration as in the traditional culture plate had a stronger apoptotic effect on a larger number of cells in microfluidic gradient environment than in a homogeneous concentration. The reason may be interactions between cells, which, after treatment with a higher concentration of polyphenol, released (as a result of degradation) another proapoptotic substances, which supported the toxic effects of the main substance on other cells.

**Figure 8:**
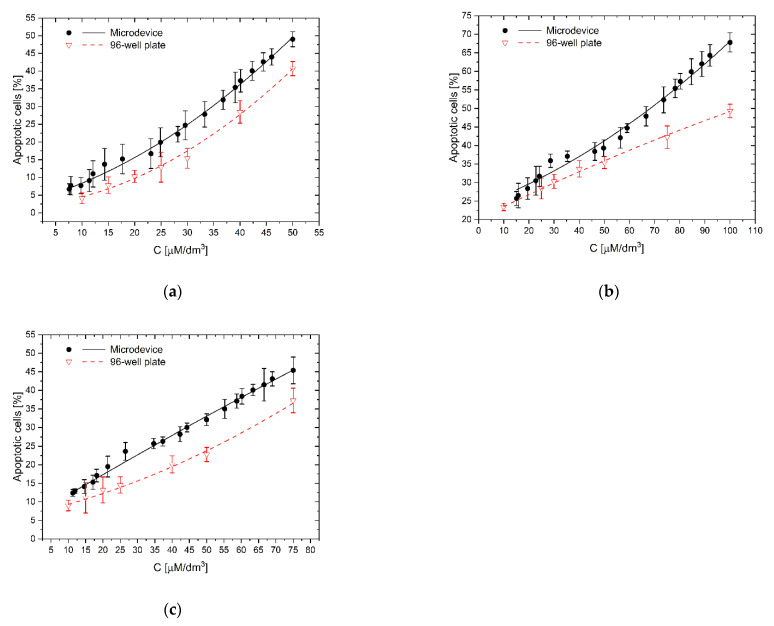
The effect of the substance concentration gradient and cellular interactions on apoptosis of LoVo cells: (**a**) curcumin; (**b**) *trans*-resveratrol; (**c**) wogonin: C–the concentration of a tested substance. Comparison of results of investigations in a gradient microsystem and a traditional 96-well culture plate.

For low curcumin concentration (10 µM/dm^3^), the apoptotic effect’s underprediction was the most significant (Figure 8a). The number of apoptotic cells in microfluidic devices reached 188% of reference value–the culture in homogeneous curcumin concentration. For higher concentrations–25 and 50 µM/dm^3^, the effect was less significant–154% and 120%, respectively, but still clearly noticed. It is interesting that for low concentrations, the direct apoptotic effect of curcumin has the same impact on cells’ viability as proapoptotic substances, like protein degradation products released to the environment from dying cells. This effect cannot be easily observed in high-volume multiwell cultures where substances released from adjacent cells are rapidly diluted. This effect was less significant for the other polyphenols, 111.0–137.5% for *trans-*resveratrol (Figure 8b), and 121.7–162.2% for wogonin (Figure 8c). For resveratrol, we have also observed, in contrast to the remaining cases, that with increasing concentration of the factor, the absolute difference in the number of apoptotic cells between the microsystem culture and the multiwell plate increases (Figure 8b). In botch remaining cases, those difference was nearly constant at the examined concentrations.

## 3. Materials and Methods

### 3.1. Cell Line

The study was carried out on the colorectal cancer line (LoVo) obtained from the ATCC collection (Manassas, VA, USA). Cells were passaged twice a week with Tryple solution for 10 min at 37 °C, then reduced by half and resuspended in culture medium or collected into a tube. Trypsin was inactivated by adding medium with serum and centrifuging for 10 min at 1000 RPM. The cells were then counted in the Bürker chamber, and appropriate densities of cells were prepared for testing in a microfluidic system or on multiwell culture plates. All cultures were grown in conditions of 5% CO_2_, 95% humidity at 37 °C.

### 3.2. Culture Medium

The cells were cultured in the medium recommended for the LoVo cell line–DMEM F12, supplemented with 10% fetal bovine serum (FBS) and appropriate antibiotics (1.25 µg/mL amphotericin B, and 100 µg/mL gentamicin).

### 3.3. Gradient Generation in Microdevice

After cells immobilization, removing of the reservoir’s covers and fluid removal from the inlet and outlet reservoirs, 14 μL of the active substance (50 μM/dm^3^ curcumin solution in culture medium, 100 μM/dm^3^ trans-resveratrol solution in culture medium or 75 μM/dm^3^ wogonin solution in culture medium) was added to the culture chamber 2 (Figure 4a).

### 3.4. Tested Compounds

The studies were performed using polyphenols with a purity of at least 98% supplied by Sigma-Aldrich: curcumin (cat. No. 78,246), *trans*-resveratrol (cat. No. R5010) and wogonin (cat. No. W0769). These compounds were dissolved in DMSO to obtain stock solutions of concentrations-22 mM for curcumin and 20 mM for trans-resveratrol and wogonin. The solutions prepared in this way were stored for up to 6 months-wogonin and *trans*-resveratrol at −20 °C, and curcumin at −80 °C. Work with *trans*-resveratrol at each stage was carried out under red light (*trans*-resveratrol instability in white light). The tested concentration of polyphenol solutions was prepared in the culture medium. In studies on multi-well plates, the following concentrations were used: 10, 15, 20, 25, 30, 40 and 50 μM for curcumin; 10, 25, 30, 40, 50, 75 and 100 μM for *trans-*resveratrol and 10, 15, 20, 25, 40, 50, 75 μM for wogonin. The indicator for measuring the gradient in a microfluidic system using a spectrophotometer was a methylene blue solution; the concentration used to set the gradient was: 10 mM.

### 3.5. Miscroscopic Evaluation of Cells Cultured in Microfluidic Device

LoVo cells were seeded into all chambers of the chip. The culture medium was changed every 24 h by pipetting fresh medium into the inlet reservoirs and removing the used one from the outlet reservoirs on the opposite side. The 96-well culture plates and microchips were placed in a CO_2_ incubator. Cells condition was evaluated using an inverted microscope. After 7 days of culture in the microsystem, LoVo cells were treated with the TrypLE solution, which is a solution with enzymes that dissociate cells. This substance is similar to trypsin but milder for cells and does not harm them in prolonged exposure. First, the liquid is replaced three times with PBS before filling the TrypLE chamber to rinse the medium with the serum. After about 1 h, most of the cells were detached from the surface, and the cell suspension is removed from the chip by exchanging the suspension with PBS through the outlet reservoir. Medium with serum was added to inactivate TrypLE, and the collected cells were centrifuged for 5 min, 1000 RPI. The cells were then seeded in a fresh medium in a conventional multiwell plate and analyzed under an EFOS FL microscope (Thermo Fisher Scientific, Waltham, MA, USA).

### 3.6. Comparison of Cell Viability in Microdevice and Multi-Well Plates

After incubation, cells in microdevices or multiwell plates for 24 and 72 h at 5% CO_2_, 95% humidity at 37 °C, the cells were evaluated using Trypan Blue dye. This dye only stains dead cells. Then, the cells were counted under light microscopy.

### 3.7. Detection of Apoptosis on Multiwell Plates

In traditional cultures, 96-wells plates were used, on which cells were seeded in a density of 10,000 per well. The culture was incubated for 24 h for cell adhesion and regeneration. The medium was removed, prepared concentrations of curcumin in a complete medium were added, and the culture was incubated for the next 24 h. Then the cells were stained with “Dead Cell Apoptosis Kit” (cat. no. V13,242, Thermo Fisher Scientific, Waltham, MA USA), containing two dyes: annexin V conjugated to FITC and propidium iodide (P.I.). The culture medium was replaced with a buffer with annexin V and left for 20 min. At RT in the dark, propidium iodide was added five minutes before the end of incubation. The photos were taken by fluorescence microscope from each well.

### 3.8. Detection of Apoptosis in a Microchip

The experiments were carried out with 5 independent repetitions, simultaneously using traditional cell cultures and a gradient microchip. The evaluation of cell apoptosis in the test compound gradient was carried out using a previously conditioned microchip with cells seeded in after a 24-h cell culture in the microchip.

**Figure 9:**
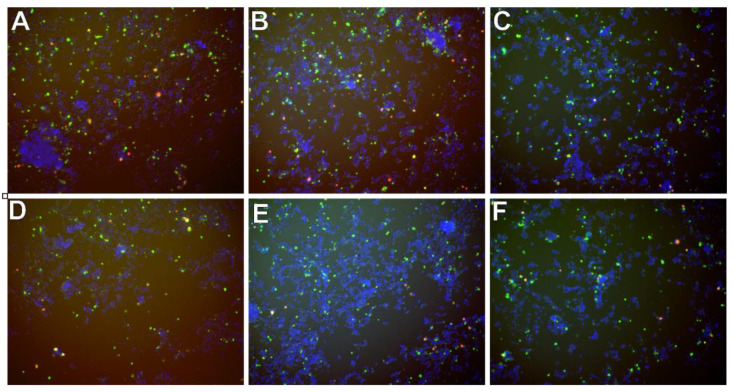
Exemplary micrographs of apoptosis evaluation after staining with Annexin-V conjugated with fluorescein (green color) and propidium iodide (red color), and all cells-after staining DAPI cell nuclei (blue color) at a concentration of 50 µM-for curcumin (**A**,**D**), trans-resveratrol (**B**,**E**) and wogonin (**C**,**F**). Photos A, B, C are taken in the chip’s CC. Photographs (**D**–**F**) in a multiwell plate.

In Figure 9 there are presented exemplary micrographs of apoptosis evaluation on microdevice (A–C) and multiwell plates (D–F). The photos show cells stained in different colors (blue, red and green). The use of DAPI allowed for the staining of all cell nuclei, both living and dead cells that are visible in blue. Necrotic cells are shown in red color and cells in apoptosis are presented in green. At this point, it is worth noting that the construction materials used to build the microsystem do not affect the quality of photos and analysis under UV light.

## 4. Conclusions

The microfluidic device for cell culture in a gradient condition used in our research allows us investigations of the influence of concentration gradients of polyphenols on colorectal adenocarcinoma cells apoptosis simply and repetitively. The small height/volume of microfluidic culture chambers and static conditions allowed for establishing the local microenvironment and maintaining undisturbed concentration profiles of naturally secreted biochemical factors from cells. We observe that these conditions significantly affect cell viability by stimulating cell apoptosis at lower concentrations of polyphenols than in traditional homogenous concentration multiwell cultures. The effect of polyphenol activity is increased by toxins arising from the decomposition of dead cells. Similar observations of curcumin’s proapoptotic effect are shown by other researchers, showing the dependence on concentration [46].

Compared to standard multiwell culture, we have not noticed an influence of diffusion flux of oxygen and nutrients on cell proliferation and viability in a chip. Also, the mild flow conditions during the convective gradient generation procedure have no visible impact on cell viability. The chip’s construction allows for cell culturing in two independent chambers providing a parallel control culture without contact with the test substance. We found that the Gompertz model best describes the cell proliferation in our gradient microfluidic chip. Performing several independent experiments (each at different times on a different chip), it was shown that the microfluidic device is characterized by a very high tolerance on human errors and stands out with high repeatability. Therefore, this device can be potentially used in pharmaceutical research to assess active substances’ properties in the future. Selected materials used in the construction of the chip avoid flaws: autofluorescence and desorption of toxic monomers. The operation of the microsystem is adapted to procedures and devices normally used in research and analytical laboratories. The medium delivery and removal from the chip can be realized by pipette use, contrary to other lab-on-a-chip flow systems, where fluids are supplied by complicated equipment (e.g., pumps, connectors, vessels etc.).

The project was aimed at checking the operation of a simple and robust microdevice that allows conducting cell cultures in a stationary (non-flow) system in a continuous gradient of the active substance and conducting research on biological processes occurring in the gradient of the substance without the need for the use of complicated flow systems that models of the structure of pathological tissues, e.g., tumors. In the next stage of research, we will develop a method of conducting tests in a dynamically changing concentration gradient. Drug levels in the body are not stable over time. Thanks to the additional chamber, which is the reference point, it is possible to determine the concentration of a substance at any point of the chamber at any time. By appropriate selection of initial concentrations in the chamber and reservoirs and the appropriate setting of the initial gradient, it should be possible to obtain a given concentration change over time. It is highly desired to construct a model system that would provide a time-dependent microenvironment similar to the conditions that we find inside the body. That should allow us to understand the dynamic influence of biochemical factors on intracellular signaling pathways, which is not achievable by classical cell cultures.

Cell cultures conducted in lab-on-a-chip systems are often used in academic research, but quite rarely in the pharmaceutical industry due to imperfections of available devices, such as the suboptimal selection of materials from which the system is made or the lack of compatibility with standard laboratory equipment, e.g., manipulators or pipetting robots and resulting from it difficulties in fabrication and use. The laser ablation of plastics, which we have used for microdevice fabrication, gave us the possibility of fabricating dozens of chips per week in a laboratory for our research purposes without costly equipment. This fabrication method even scales up to industrial-scale production, so there is a straightforward way to launch the market’s chip. We hope that lab-on-a-chip systems will be introduced on a larger scale in the pharmaceutical industry in the near future, as they have a large potential to shorten the time and lower the costs of new drug development.

## 5. Patents

(1) Szafran Roman, Kazimierz Gąsiorowski, Katarzyna Gębczak, Benita Wiatrak, Microfluidic device for cell culture in gradient of bioactive substance, WO2018106132 (A1), 2018.
(2) Szafran Roman, Kazimierz Gąsiorowski, Katarzyna Gębczak, Benita Wiatrak, Microfluidal device for growing cell culture in a gradient of bioactive substance PL419754 (A1), 2016.
(3) Szafran Roman, Method for manufacturing micro fluidised bed device for growing cell culture in a gradient of bioactive substance, PL419758 (A1), 2016.
(4) Szafran Roman, Kazimierz Gąsiorowski, Katarzyna Gębczak, Benita Wiatrak, Method for producing stable active substance concentration gradient in the cell culture microsystems, PL419813 (A1), 2016.

## Data Availability

Not applicable.

## Sample Availability

Not available.
